# Associations between multimorbidity burden and objective and patient-reported sleep outcomes among people with HIV

**DOI:** 10.1097/QAD.0000000000004073

**Published:** 2024-11-28

**Authors:** Luxsena Sukumaran, Caroline A. Sabin, Ken M. Kunisaki, Nicki Doyle, Frank A. Post, Jaime Vera, Patrick W.G. Mallon, Memory Sachikonye, Marta Boffito, Jane Anderson, Alan Winston

**Affiliations:** a Institute for Global Health, University College London; b National Institute for Health Research (NIHR) Health Protection Research Unit (HPRU) in Blood-borne and Sexually Transmitted Infections at University College London, London, UK; c Minneapolis Veterans Affairs Health Care System; d University of Minnesota, Minneapolis, Minnesota, USA; e Department of Infectious Disease, Imperial College London; f King's College Hospital NHS Foundation Trust, London; g Brighton and Sussex Medical School, Brighton, UK; h University College Dublin, Dublin, Ireland; i UK Community Advisory Board (UK-CAB); j Chelsea and Westminster Healthcare NHS Foundation Trust; k Homerton University Hospital, London, UK.

**Keywords:** comorbidity, HIV, multimorbidity, multimorbidity patterns, sleep

## Abstract

**Background:**

We aimed to provide insights into the effects of comorbidities on sleep health in people with HIV by assessing associations between multimorbidity patterns and sleep outcomes in the Pharmacokinetic and clinical Observations in PeoPle over fiftY (POPPY) sub-study.

**Methods:**

Principal component analysis identified six multimorbidity patterns among participants with HIV (*n* = 1073) at baseline: cardiovascular diseases (CVDs), sexually transmitted diseases, metabolic, mental/joint, neurological and cancer/other. Burden *z* scores were calculated for each individual/pattern. A subset of 478 participants completed sleep assessments at follow-up, including questionnaires [Insomnia Severity Index (ISI), Patient-Reported Outcomes Measurement Information System (PROMIS) Sleep Disturbance (SD) and Sleep Related Impairment (SRI)] and overnight oximetry [4% oxygen desaturation index (ODI) and percentage of time with oxygen saturation (SpO_2_) <90%). Multivariable regression assessed associations between burden *z* scores and sleep measures.

**Results:**

Amongst 309 participants [median (interquartile range) age 53 (47–59) years], 21% had insomnia (ISI ≥15). Higher *Mental/Joint z* scores were associated with increased odds of insomnia [aOR 1.06 (95% CI 1.03–1.09)] and worse PROMIS-SRI [1.34 (1.22–1.48)] and PROMIS-SD [1.27 (1.16–1.39)] scores. Higher metabolic and neurological *z* scores were associated with worse PROMIS-SRI scores (*P* < 0.01). Higher CVDs *z* scores were associated with worse ISI and PROMIS-SRI scores, and a higher percentage of time with SpO_2_ below 90% (all *P*'s < 0.01).

**Conclusion:**

This study is among the first to describe specific multimorbidity patterns linked to poorer sleep outcomes in people with HIV. Findings suggest the need for targeted sleep interventions based on multimorbidity profiles, which may mitigate broader health risks associated with poor sleep.

## Introduction

With widespread access to antiretroviral therapy (ART), HIV infection has become a manageable chronic condition with a life expectancy that is comparable with that of people without HIV [1,2]. Consequently, the focus on HIV has now shifted from survival to the increased burden and management of age-associated comorbidities, including cardiovascular disease (CVD), chronic obstructive pulmonary disease, type 2 diabetes and chronic kidney disease [3–5].

Sleep disorders such as insomnia, obstructive sleep apnoea (OSA), and restless leg syndrome (RLS) are also increasingly common among people with HIV [6,7], with a meta-analysis reporting that the prevalence of sleep disturbances was around 58% in those living with HIV, compared with 10–35% in the general population [8]. People with HIV may be particularly vulnerable to sleep disorders due to multiple contributing factors, including psychosocial challenges (e.g. depression, anxiety and stigma), lifestyle habits, HIV-mediated immune and neuronal dysfunction, and adverse consequences of ART [9–12]. Sleep is an important determinant of physiological function [13], and several studies have shown that the negative health consequences of poor sleep include impaired daytime functioning (e.g. decreased concentration, daytime sleepiness, memory decline), poorer health-related quality of life and a higher incidence of comorbidities (e.g. diabetes mellitus, cognitive dysfunction and psychiatric disorders) [14–16]. In the context of HIV infection, sleep disorders may also increase the risk of nonadherence to ART, treatment failure, psychological symptoms and the severity of HIV-related symptoms [17,18].

Although individuals living with HIV are experiencing a rising prevalence of both multimorbidity and sleep problems, the complex interplay between these two factors is not fully understood. Emerging data from HIV cohorts have suggested that the presence of sleep disorders increases the risk of comorbidities, such as cardiovascular disease (e.g. myocardial infarction, stroke and heart failure), mental health disorders (e.g. depression and anxiety), cognitive decline and pain [19]. However, another possibility is that the presence of multiple health conditions may exacerbate sleep problems, either directly through shared pathophysiological mechanisms or indirectly through, say, psychological distress or adverse effects from medications. Some studies have suggested that a bidirectional relationship may exist between sleep and psychiatric disorders (e.g. depression and anxiety) in people with HIV [20,21]. However, the aforementioned studies have explored the associations between individual comorbidities and, often single, sleep disorders among people with HIV. Increasingly, data-driven approaches have been used to understand the most common patterns of morbidities (multimorbidity) that are more likely to co-occur within the same individual due to similar pathophysiological mechanisms and/or shared risk factors [22]. In turn, we can gain a better understanding of the causes underlying specific patterns, as well as the complex interplay between conditions on outcomes such as sleep quality/health.

Therefore, in the present study, we investigated associations between data-driven multimorbidity patterns and a range of both objective and patient-reported sleep outcomes among people with HIV enrolled in the multicentre Pharmacokinetic and clinical Observations in PeoPle over fiftY (POPPY) Sleep sub-study in the United Kingdom and Ireland.

## Methods

### Study participants and procedures

The POPPY study is a prospective cohort study of people with HIV and HIV-negative individuals from the United Kingdom and Ireland. Characteristics and eligibility criteria of the POPPY cohort are detailed elsewhere [23]. Briefly, the study recruited three groups of individuals between April 2013 to January 2016: people with HIV aged at least 50 years (older people with HIV), people with HIV aged 18–49 years (younger people with HIV), and HIV-negative controls aged at least 50 years who were frequency-matched to the older people with HIV on gender, ethnicity, sexual orientation, and location (in or out of London). At the study visits, information on sociodemographic characteristics, comorbidities, and laboratory measurements was collected by trained clinical staff, and through data linkage with the UK Collaborative HIV Cohort (UK CHIC) study and the Mater Misericordiae University Hospital (MMUH) Infectious Diseases (ID) Cohort in Dublin.

A subset of POPPY study participants was recruited into the nested POPPY-Sleep sub-study, independent of any existing sleep symptoms [7]. Inclusion criteria were the ability to wear a fingertip oximetry device and wrist actigraph for a week, as well as adherence to study procedures (determined by investigator's judgement). Participants had a single study visit between March 2017 and July 2018, where they completed questionnaires on sleep quality, symptoms of sleep disorders and sleep medical history, and underwent detailed assessment of anthropometric measurements. This was followed by in-home assessments including a daily sleep diary, actigraph and oximetry measurements. All participants provided written informed consent, and ethical approval was granted by the UK National Research Ethics Service (NRES; Fulham London, UK number 12/LO/1409) for the POPPY study and the UK Health Research Authority & Research Ethics Committee (number 16/LO/2175) for the POPPY-Sleep sub-study.

### Data on comorbidities

As mentioned previously, participants self-reported the presence or absence of comorbidities during structured interviews conducted by trained research staff at the main POPPY baseline visit, and wherever possible, this information was confirmed using concomitant medication (nonantiretroviral) and healthcare utilisation data (e.g. visits to general practitioners, hospitals, psychiatrists/psychologists, and specialists). Participants were asked about 52 health conditions listed in the questionnaire, and to add any other conditions experienced (using free-text questions). For the present analysis, we considered 70 comorbidities (from 19 organ system/pathogenic groups) with a prevalence at least 1.5% in the study population (Supplementary Table 1).

### Sleep outcomes

A subset of participants completed sleep questionnaires at the POPPY Sleep substudy visit when returning the actigraphy/oximetry devices, including the Insomnia Severity Index (ISI) and the Patient-Reported Outcomes Measurement Information System (PROMIS) Sleep Disturbance (-SD) and Sleep Related Impairment (-SRI) questionnaires. For the present analysis, continuous ISI scores were examined, as well as a binary classification (ISI score ≥15) of insomnia. Raw scores from the PROMIS questionnaires were transformed into *T* scores, where a population mean score is 50 and standard deviation is 10. Higher *T* scores represent greater sleep disturbance or sleep-related impairment.

Oximetry measurements were recorded from in-home overnight oximetry using a battery-powered, wrist-worn overnight fingertip pulse oximetry device (WristOx 3150; Nonin Medical, Plymouth, Minnesota, USA). Oximetry data recordings were examined to identify periods of likely sleep period and artifact. These data were then processed to generate oxygen saturation metrics, including Oxygen Desaturation Index (ODI; 4% desaturation) and percentage of time with oxygen saturation (SpO_2_) below 90% (i.e. time hypoxic, or sleep-related hypoxia/hypoxemia). Further information on the data collection procedures for oximetry data have been described in greater detail elsewhere [7].

### Statistical analysis

As described previously [24,25], we used Principal Component Analysis (PCA) to identify patterns of multimorbidity. Briefly, PCA is a data reduction technique that reduces the dimensionality of a dataset (with a large number of interrelated variables) whilst retaining the maximum amount of variance among the original variables. Pairwise associations between the 70 comorbidities, reported by participants at baseline, were assessed using Somers’ *D* statistic, as proposed by Ng *et al.*[26]. PCA was then applied to the matrix containing the pairwise associations to identify a smaller set of principal components, which can be interpreted as multimorbidity patterns, that is, nonrandom groups of comorbidities that are frequently associated with each other. In addition, an *oblimin* rotation was used to allow patterns to be associated with each other and thus, multiple patterns to be present within the same individual. The optimal number of principal components or patterns was determined using scree plot and the very simple structure (VSS) criterion. Given the lack of established empirical guidelines, we evaluated various correlation/loading thresholds (i.e. 0.10, 0.20, 0.25, 0.30, 0.40) to determine which comorbidities should be included in each pattern. A threshold of at least 0.25 was used to determine comorbidities that were significantly associated with each pattern. This threshold was selected based on discussions with clinicians as comorbidities that met this threshold were found to be clinically associated with one another, that is, having shared risk factors and/or pathophysiological mechanisms as the other comorbidities in the same pattern. Morbidity burden scores were then calculated for each participant/pattern using the main POPPY follow-up visit data on the presence/absence of the comorbidities that had a correlation of at least 0.25 with the corresponding pattern. Scores were then standardized and multiplied by 10 (0.1 SD) to ease interpretation, with scores greater than 0 reflecting higher morbidity burden compared with the study sample mean. For the main analysis, we examined the associations of sleep outcomes with morbidity burden *z* scores from the most recent POPPY visit (follow-up visit) prior to the POPPY SLEEP substudy visit. Therefore, participants who only attended the main POPPY baseline visit and did not return for the follow-up visit were excluded.

The associations of morbidity burden *z* scores with ISI scores, PROMIS-SD and PROMIS-SRI T scores, ODI and percentage of time with SpO_2_ below 90% were assessed using linear regression. Insomnia (defined as an ISI score >15) associations were assessed using logistic regression. Models were adjusted for the following covariates, identified *a priori* based on existing literature, that were collected at the POPPY baseline visit (when comorbidities were assessed): age, sex, race, obesity (BMI >30 kg/m^2^), sex between men, smoking status, alcohol use, history of injection drug use and recreational drug use, with estimates expressed as adjusted odds ratios (aOR) or adjusted beta estimates with 95% confidence intervals (95% CI). A significance level of *P* less than 0.05 guided statistical interpretation. Three exploratory post hoc analyses were also conducted: associations between morbidity burden *z* scores and sleep outcomes while additionally adjusting for depressive symptoms using PHQ-9 scores (*n* = 284); analysis to examine whether associations with changes in burden *z* scores (differences between morbidity *z*- scores at the POPPY follow-up and baseline visits) and the sleep outcomes of interest were consistent with the findings from the main analyses; and analysis to explore whether the associations between morbidity burden *z* scores and sleep outcomes differed when a stricter PCA threshold of at least 0.40 was used to generate burden *z* scores.

The analyses were performed in all POPPY participants with HIV using R V4.2.4 (R Foundation for Statistical Computing, Vienna, Austria).

## Results

### Characteristics of study participants

Of the 357 participants with HIV enrolled in the POPPY-Sleep substudy, 309 participants (86.6%) had comorbidity data that preceded their sleep visit and complete sleep data on the outcomes of interest. We compared baseline characteristics between the final analytic sample and the overall POPPY Study population and found no large differences between the two, suggesting that the final sample is representative of the broader study cohort (Supplementary Table 2). The sociodemographic, lifestyle and HIV-related characteristics of included participants are summarized in Table 1. Participants were predominantly male (86.1%), of white ethnicity (88.7%), MSM (78.6%) with a median (IQR) age of 53 (47–59) years. The majority of participants were on ART (97.1%) and had an undetectable viral load (<50 copies/ml; 92.2%). The median (IQR) CD4^+^ T-cell count and years since HIV diagnosis were 602 (470–792) cells/μl and 14.6 (8.1–21.4) years, respectively. The median (IQR) time between the POPPY enrollment (baseline visit) and POPPY-Sleep substudy visit was 39 (31–45) months (the shortest and longest intervals were 21 and 60 months, respectively). The median (IQR) time from the POPPY follow-up visit to POPPY-Sleep substudy visit was 10 (4–17) months (range: 0–34 months).

**Table 1:** Baseline sociodemographic, lifestyle and HIV-related characteristics among all POPPY SLEEP substudy participants with HIV and comorbidity profile information (*n* = 309).

Characteristic [*n* (%)] or median (IQR)	Total (*n* = 309)
Age (years)	53 (47–59)
Gender	
Male	266 (86.1)
Female	43 (13.9)
Ethnicity	
Black-African	35 (11.3)
White	274 (88.7)
Sexual orientation	
MSM	243 (78.6)
Heterosexual	66 (21.4)
BMI (kg/m^2^)	24.8 (22.7–28.2)
Smoking status	
Never	118 (38.2)
Past	112 (36.3)
Current	79 (25.6)
Alcohol use	
Never	16 (5.2)
Past	44 (14.2)
Current	249 (80.6)
PHQ-9 score	5 (1–10)
Undetectable viral load (HIV RNA <50 copies/ml)	285 (92.2)
Years since HIV diagnosis	14.6 (8.1–21.4)
On ART	300 (97.1)
Prior AIDS event	81 (26.2)
Time between study visits (months)	
POPPY Baseline and Sleep visit	39 (31–45)
POPPY Follow up and Sleep visit	10 (4–17)

IQR, interquartile range; POPPY, Pharmacokinetic and clinical Observations in PeoPle over fiftY.

### Multimorbidity patterns

Using PCA, six multimorbidity patterns were identified in all POPPY participants with HIV using baseline data, accounting for 23.3% of the total variance in the 70 comorbidities: cardiovascular diseases (CVDs); sexually transmitted diseases (STDs); metabolic; mental/joint; neurological; and cancer/other. The comorbidities with a correlation (or loading) at least 0.25 with each distinguished pattern are reported in Table 2.

**Table 2:** Multimorbidity patterns identified using principal component analysis in all POPPY participants with HIV (*n* = 1073) at the baseline visit.

PC (% of variance explained)	Label	Comorbidities with correlation ≥0.25 (correlation with PC)
1 (6.5%)	CVDs	CABG/PCTA (0.68), heart failure (0.62), hypertension (0.70), IHD (0.72), myocardial infarction (0.69), peripheral vascular disease (0.46), renal problems (0.40), Dyslipidemia (0.34)
2 (4.5%)	STDs	gonorrhoea (0.78), chlamydia (0.68), LGV (0.64), syphilis (0.64), HSV (0.48), hepatitis C (0.36), hepatitis A (0.35)
3 (3.8%)	Metabolic	Peripheral neuropathy (0.57), type II diabetes (0.57), hypothyroidism (0.46), dyslipidemia (0.45), pruritis (0.41), prostate dysfunction (0.31), KS (0.30), PCP (0.30), CMV (0.28), eye problems (0.26), hepatitis B (0.26), skin cancer (0.26), urinary incontinence (0.25)
4 (3.1%)	Mental/joint	Clinical depression (0.75), anxiety/panic attacks (0.50), joint inflammation/arthritis (0.45), joint replacement (0.45), sleeping problems (0.39), bowel disorders (0.39), asthma/COPD (0.34), lipodystrophy (0.34), eczema (0.28), gastrointestinal reflux (0.27), hypogonadism (0.25)
5 (2.9%)	Neurological	Dizziness/vertigo (0.61), encephalitis (0.60), loss of consciousness (0.39), pruritus (0.32), other AIDS (0.28), migraines/headaches (0.28), psychosis (0.27), aches and pains (0.27)
6 (2.6%)	Cancer/other	Haematological cancer (0.64), hernia (0.45), osteopenia/osteoporosis (0.44), AIDS-related cancer (0.43), PCP (0.32), CMV (0.29), PVD (0.28), DVT (0.27), HSV (0.26), other AIDS (0.25)

CABG/PCTA, coronary-artery bypass grafting/percutaneous transluminal coronary angioplasty; CMV, cytomegalovirus; CVDs, cardiovascular diseases; DVT, deep vein thrombosis; HSV, herpes simplex virus; IHD, ischemic heart disease; KS, Kaposi sarcoma; LGV, lymphogranuloma venereum; PCP, pneumocystis pneumonia; PVD, peripheral vascular disease; STDs, sexually transmitted diseases.

**Fig. 1:**
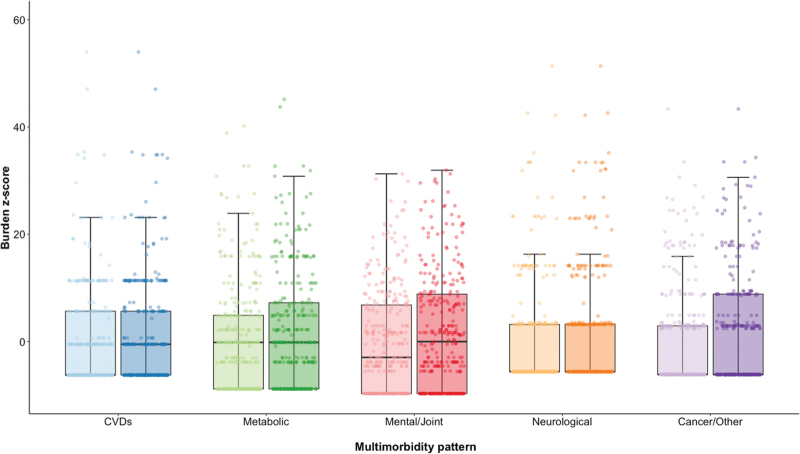
Distribution of participants morbidity burden z-scores (generated using a PCA threshold > 0.25) across the multimorbidity patterns at both the main POPPY baseline (lighter colours) and follow-up (darker colours) visit.

Morbidity burden scores were then generated for each participant (who had returned for a follow-up visit) and each pattern (excluding the STDs pattern as these comorbidities were of acute nature) at baseline as well as at the follow-up visit, 3–5 years later. Median (IQR) burden *z* scores at follow-up (in decreasing order) were −0.02 (−9.73 to 8.83), −0.17 (−8.86 to 7.22), −0.52 (−6.25 to 5.64), −5.64 (−5.64 to 3.24), −6.16 (−6.16 to 8.84) for the mental/joint, metabolic, CVDs, neurological, cancer/other *z* scores, respectively (Fig. 1). Median (IQR) burden *z* scores at baseline (in decreasing order) were −0.17 (−8.86 to 4.88), −2.97 (−9.73 to 6.80), −5.64 (−5.64 to 3.18), −6.16 (−6.16 to 2.92), −6.25 (−6.25 to 5.64) for the metabolic, mental/joint, neurological, cancer/other and CVDs *z* scores, respectively (Fig. 1).

### Associations with sleep outcomes

Overall, 66 (21.4%) of the 309 participants met the criteria for insomnia (ISI >15). Median (IQR) scores for ISI, PROMIS-SRI and PROMIS-SD were 8 (4–14), 50.3 (43.6–57.2), and 52.2 (45.5–57.3), respectively. Median ODI was 3.0 (1.3–6.3) and percentage of time with SpO_2_ below 90% was 0.3 (0.0–2.5).

In the univariate models, a 0.10 standard deviation increase in metabolic, mental/joint and neurological *z* scores were significantly associated with higher PROMIS-SRI and PROMIS-SD *T* scores (Table 3). In addition, a higher mental/joint and neurological *z* score was also associated with a higher ISI score [mental/joint: 1.18 (1.11–1.26); neurological: 1.08 (1.01–1.15)]. in terms of the oximetry measures, higher CVDs [1.14 (1.07–1.23)] and metabolic [1.15 (1.08–1.23)] *z*-scores were significantly associated with a higher ODI. furthermore, the CVDs, metabolic and cancer/other *z* scores were associated with a higher percentage of time with SPO_2_ below 90% [CVDs: 1.38 (1.19–1.60); metabolic: 1.35 (1.18–1.56); cancer/other: 1.23 (1.05–1.42)].

**Table 3:** Univariate and multivariable models showing the associations of morbidity burden *z* scores with sleep outcomes using logistic/linear regression models; reported estimates show the estimated change in the odds ratio (insomnia, ISI ≥15) and score (ISI, PROMIS-SRI, PROMIS-SD, ODI and percentage of time with SpO_2_ below 90%) associated with a 0.1 standard deviation increase in each burden *z* score.

	Sleep outcomes					
Morbidity burden *z* scores	Insomnia (>15 ISI score) OR (95% confidence interval)	Insomnia (cont.) *β* (95% confidence interval)	Sleep-related impairment *T* score *β* (95% confidence interval)	Sleep disturbance *T* score *β* (95% confidence interval)	Oxygen Desaturation Index (4% desaturation) (events per hour) *β* (95% confidence interval)	Percentage of time with SpO_2_ below 90% *β* (95% confidence interval)
CVDs						
Unadjusted	1.01 (0.99–1.04)	1.05 (0.98–1.13)	1.06 (0.96–1.17)	1.02 (0.93–1.12)	1.14 (1.07–1.23)	1.38 (1.19–1.60)
Adjusted	1.02 (0.99–1.05)	1.09 (1.00–1.18)	1.12 (1.00–1.25)	1.05 (0.95–1.17)	1.08 (1.00–1.17)	1.25 (1.06–1.47)
Metabolic						
Unadjusted	1.00 (0.97–1.02)	1.04 (0.98–1.12)	1.11 (1.01–1.22)	1.11 (1.01–1.21)	1.15 (1.08–1.23)	1.35 (1.18–1.56)
Adjusted	1.00 (0.97–1.03)	1.07 (0.99–1.15)	1.16 (1.05–1.29	1.13 (1.02–1.24)	1.12 (1.04–1.20)	1.24 (1.06–1.44)
Mental/joint						
Unadjusted	1.05 (1.03–1.08)	1.18 (1.11–1.26)	1.34 (1.23–1.47)	1.28 (1.18–1.39)	1.01 (0.94–1.08)	1.10 (0.95–1.27)
Adjusted	1.06 (1.03–1.09)	1.20 (1.12–1.28)	1.34 (1.22–1.48)	1.27 (1.16–1.39)	1.01 (0.94–1.08)	1.09 (0.94–1.26)
Neurological						
Unadjusted	1.02 (1.00–1.04)	1.08 (1.01–1.15)	1.21 (1.11–1.33)	1.10 (1.01–1.20)	1.03 (0.96–1.10)	0.88 (0.77–1.01)
Adjusted	1.01 (0.99–1.04)	1.07 (1.00–1.15)	1.20 (1.09–1.32)	1.09 (1.00–1.19)	1.02 (0.96–1.09)	0.89 (0.77–1.02)
Cancer/other												
Unadjusted	0.97 (0.94–1.00)	0.97 (0.90–1.04)	0.99 (0.89–1.09)	1.02 (0.92–1.12)	1.01 (0.94–1.09)	1.23 (1.05–1.42)
Adjusted	0.97 (0.94–1.00)	0.96 (0.89–1.03)	0.98 (0.88–1.09)	0.98 (0.89–1.08)	1.00 (0.93–1.08)	1.15 (0.98–1.34)

CVDs, cardiovascular disease; ISI, Insomnia Severity Index (ISI), PROMIS-SD, Patient-Reported Outcomes Measurement Information System-Sleep Disturbance; ODI, oxygen desaturation index.

After adjusting for confounders of age, sex, race, obesity, sex between men, smoking status, alcohol use, history of injection drug use and recreational drug use, a 0.10 SD increase in the CVD *z* score was associated with a poorer ISI [1.09 (1.00–1.18)] and PROMIS-SRI [1.12 (1.00–1.25)] score and a higher percentage of time with SpO_2_ below 90% [1.25 (1.06–1.47)} (Table 3). The significant univariable associations observed with the metabolic and mental/joint *z* scores remained after adjustment for confounders. Higher neurological *z* scores were still associated with worse PROMIS-SRI scores [1.20 (1.09–1.32), *P* < 0.0001] but were no longer significantly associated with ISI [1.07 (1.00–1.15), *P* = 0.05] and PROMIS-SD scores [1.09 (1.00–1.19) *P* = 0.05]. In contrast, the cancer/other *z* score was not significantly associated with any of the sleep outcomes after adjustment for covariates.

### Exploratory post hoc analyses

After adjusting for the aforementioned confounders and PHQ-9 scores, some of the associations between burden *z* scores and sleep outcomes were comparable in magnitude to the previous adjusted analyses, but were no longer statistically significant due to wider CIs (Supplementary Table 3). When examining the relationship between changes in burden *z* scores and sleep outcomes, only mental/joint *z* scores showed significant associations. These were linked to increased odds of insomnia [1.09 (1.02–1.17)], as well as worse scores in ISI [1.37 (1.13–1.67)], PROMIS-SRI [1.78 (1.35–2.33)], and PROMIS-SD [1.48 (1.14–1.93)] scores after adjustment (Supplementary Table 4).

Fewer comorbidities were linked to multimorbidity patterns when a stricter PCA threshold (≥0.40) was used (Supplementary Table 5). When analysing *z* scores based on this threshold, we found that a number of associations with sleep outcomes, that were previously significant with a PCA threshold greater than 0.25, had become insignificant (Supplementary Table 6). However, the direction of these associations remained consistent across both thresholds, though slightly weaker. Therefore, while the trends in associations were similar with both thresholds, associations were found to be stronger with a lower PCA threshold (i.e. ≥0.25).

## Discussion

This is the first study, to our knowledge, that has assessed the relationship between multimorbidity patterns and a range of objective and patient-reported sleep outcomes in a large multicentre cohort of people with HIV. The clustering of conditions within the multimorbidity patterns does not strictly follow traditional diagnostic categories but instead reflects underlying shared pathophysiological mechanisms among the comorbidities. For example, the co-occurrence of mental health issues with joint conditions may indicate a broader chronic illness syndrome where physical health affects mental well being (and vice versa). Moreover, the inclusion of psychosis in the ‘Neurological’ pattern highlights potential neurocognitive impacts on mental health, while peripheral neuropathy in the ’Metabolic’ pattern suggests a link between metabolic dysregulation and nerve damage. We found that certain multimorbidity patterns (CVDs, metabolic, mental/joint and neurological) were strongly associated with worsened self-reported (PROMIS-SRI, PROMIS-SD, insomnia) and objective (ODI and percentage of time with SpO_2_ below 90%) sleep measures.

Several cross-sectional studies have also shown that individuals with multimorbidity (measured using a simple count of health conditions) have a higher risk for obstructive sleep apnoea and an increased odds of sleep problems [27–29]. Our findings are consistent with these studies, demonstrating associations between specific multimorbidity patterns and various sleep outcomes in people with HIV. Mechanisms that may underlie the observed associations between multimorbidity and sleep outcomes may include psychological burden/distress (anxiety and stress) and pain due to symptom burden, treatment complexity/costs and increased functional impairment, which in turn, may exacerbate sleep problems [30–32]. Other mechanisms may include the presence of sleep-disordered breathing in certain conditions such as diabetes, chronic lung disease, and stroke [33,34]. Multimorbidity has also been associated with increased levels of sedentary time [35], which has been implicated in poor sleep. Previous research suggests that may be explained by an increased risk of depression and metabolic syndrome, and greater exposure to LED-backlit TV screens [36]. Furthermore, sleep problems, in turn, may also increase the risk and/or burden of multimorbidity through mechanisms such as impairment in the hypothalamic–pituitary–adrenal (HPA) axis or inflammation, which may consequently lead to a detrimental cycle where comorbidities and sleep problems may exacerbate each other [29]. This is supported by Sindi *et al.*[37] who demonstrated that moderate to severe sleep disturbances were associated with a faster accumulation of chronic diseases during a 9-year follow-up. This underscores the possibility of early detection and treatment of sleep problems as a potential strategy to mitigate the burden of multimorbidity. More specifically, our work suggests that sleep interventions might have a role in preserving cardiac, metabolic, neurologic and mental health in people with HIV. However, it is important to recognize that these interventions should not be considered as isolated solutions but instead as a part of a comprehensive care strategy. The complexities of multimorbidity requires a multidisciplinary approach, and further efficacy and effectiveness studies are needed to determine the potential of sleep interventions alongside other therapeutic strategies in this population.

The existing evidence among HIV populations is scarce, but emerging studies have shown a complex and interrelated relationship between sleep and psychiatric comorbidities in people with HIV [38]. For instance, both depression and sleep problems have been associated with poorer health-related quality of life, and anxiety has been associated with more sleep problems in people with HIV [39]. This is consistent with our findings where we observed that the mental health/joint pattern had the strongest associations with adverse sleep outcomes. These associations may be partly explained by psychological distress associated with the fear of stigma and social isolation experienced by people with HIV [40–42].

The strengths of our study include the use of validated instruments to define sleep outcomes and the assessment of multiple dimensions of sleep, including objective measures and patient-reported perceptions of sleep health. Nevertheless, there are some limitations to our study. First, given the cross-sectional nature of our study, causality or temporal associations between multimorbidity patterns and sleep outcomes cannot be established. Although we explored associations between changes in burden scores and sleep outcomes, these findings were based on historic changes (baseline data on comorbidities preceded the sleep study visit, with a median of 3.3 years), and therefore, should be interpreted with caution. The time interval between the follow-up visit and the sleep sub-study visit also varied across participants, which could potentially influence the observed associations. Moreover, the follow-up study visit preceded the sleep study visit, and reported sleep problems may have existed for years prior to the sleep study visit. Unfortunately, this cannot be confirmed as data on the onset of sleep problems was not collected. As a result, whether a bidirectional relationship exists between multimorbidity and sleep could not be explored. However, the POPPY study has two additional study visits planned for data collection, which will allow us to explore how changes in multimorbidity patterns over time may relate to sleep outcomes as more longitudinal data become available. Second, information on comorbidities was self-reported by participants, and thus, study findings may be affected by recall bias. However, wherever possible, this information was checked against healthcare utilization and concomitant medication data. Our sample's insomnia prevalence of 21% may appear lower than that reported in other studies among people with HIV [8], but likely reflects differences in sample demographics, assessment methods, and variations in insomnia definitions used across studies. For example, we used a validated insomnia questionnaire rather than self-report and we used a threshold that correlated to a clinical diagnosis of moderate to severe insomnia, rather than mild or subthreshold insomnia. Third, the identified multimorbidity patterns account for only 23.3% of the total comorbidity variance. In the present study, the complexity of multimorbidity was captured by examining interactions among a diverse array of relevant conditions, making it challenging to capture a large amount of variation with a limited number of patterns. Although increasing the number of patterns would improve the explained variance, it could also compromise interpretability and yield clinically irrelevant clusters. Moreover, we used robust methods to determine the optimal number of patterns, providing valuable insights into the clustering of comorbidities within our study population. Fourth, the burden *z* scores were derived from the mean of our sample. Although this approach allows us to evaluate the impact of multimorbidity on sleep outcomes within this specific population, it is important to acknowledge that *z* scores greater than zero may reflect elevation from a high baseline rather than normative expectations. This approach may limit the generalizability of our findings to the broader population. Future studies should consider incorporating comparator groups to enhance the interpretability of such metrics and their clinical relevance. Fifth, our findings may have been affected by residual confounding as we were unable to adjust for all potential confounders, such as physical activity status, life stressor burden, social support and coping strategies. Finally, our cohort of people with HIV comprised predominately of men of white ethnicity with longstanding, well controlled HIV infection in a high-income region of the world. Therefore, our findings may not be generalizable to other HIV populations with different characteristics such as women, other ethnicities, those with later/advanced HIV disease and those living in low-to-middle income regions. Further work in this area is needed to determine whether our findings are consistent across other subgroups.

In conclusion, we identified strong associations between certain multimorbidity patterns, including CVDs, metabolic, mental/joint and neurological, and sleep outcomes among people with HIV. Our work supports the importance of early care provider screening for sleep symptoms and highlight the need to further investigate whether timely interventions are crucial for optimizing the health and quality of life of older individuals living with HIV. By addressing sleep issues proactively, healthcare providers can potentially mitigate the risks associated with multimorbidity. Furthermore, future longitudinal studies should assess temporal associations and examine the bidirectional relationship between multimorbidity and sleep in greater detail.

## Acknowledgements

POPPY Management Team: Marta Boffito, Patrick Mallon, Frank Post, Caroline Sabin, Memory Sachikonye, Alan Winston, Christina Prechtl. POPPY Scientific Steering Committee: Jane Anderson, David Asboe, Marta Boffito, Lucy Garvey, Patrick Mallon, Frank Post, Anton Pozniak, Caroline Sabin, Memory Sachikonye, Jaime Vera, Ian Williams, Alan Winston. POPPY Sites and Trials Unit: Caldecot Centre, King's College Hospital (Frank Post, Lucy Campbell, Selin Yurdakul, Sara Okumu, Louise Pollard, Beatriz Santana Suárez, Luella Hanbury) Department of Infection and Population Health, UCL (Ian Williams, Damilola Otiko, Laura Phillips, Rosanna Laverick, Michelle Beynon, Anna-Lena Salz, Abigail Severn, Michelle Beynon, Gosala Gopalakrishnan) Clinical Research Facility Allan at St Mary's, Brighton and Sussex University Hospital (Martin Fisher, Amanda Clarke, Jaime Vera, Andrew Bexley, Celia Richardson, Sarah Kirk, Rebecca Gleig, Leigh Greenland) HIV Molecular Research Group, School of Medicine, UCD (Patrick Mallon, Alan Macken, Bijan Ghavani-Kia, Joanne Maher, Maria Byrne, Ailbhe Flaherty, Aoife McDermott, Riya Negi, Alejandro Garcia-Leon and Aoife Cotter) Homerton Sexual Health Services, Homerton University Hospital (Jane Anderson, Sifiso Mguni, Rebecca Clark, Rhiannon Nevin-Dolan, Sambasivarao Pelluri, Tracey Fong) Ian Charleson Day Centre, Royal Free Hospital (Margaret Johnson, Nnenna Ngwu, Nargis Hemat, Anne Carroll, Sabine Kinloch, Mike Youle, Sara Madge and Katie Spears) Imperial Clinical Trials Unit, Imperial College London (Daphne Babalis, Jodi Meyerowitz, Christina Prechtl) St. Mary's Hospital London, Imperial College Healthcare NHS Trust (Alan Winston, Lucy Garvey, Merle Henderson, Claire Peterson, Wilbert Ayap, Allan Lisenco and Ian McGuinness) St Stephen's Centre, Chelsea and Westminster Hospital (Marta Boffito, David Asboe, Anton Pozniak, Margherita Bracchi, Nicole Pagani, Maddalena Cerrone, Daniel Bradshaw, Francesca Ferretti, Chris Higgs, Elisha Seah, Stephen Fletcher, Michelle Anthonipillai, Ashley Moyes, Katie Deats, Irtiza Syed, Clive Matthews, Peter Fernando, Cherry Colcol). POPPY methodology/statistics: Caroline Sabin, Hajra Okhai, Luxsena Sukumaran. NIHR HPRU Steering Committee: Professor C.A. Sabin (HPRU Director), Dr J. Saunders (UKHSA Lead), Professor C. Mercer, Dr H. Mohammed, Professor G. Rait, Dr R. Simmons, Professor W. Rosenberg, Dr T. Mbisa, Professor R. Raine, Dr S. Mandal, Dr R. Yu, Dr S. Ijaz, Dr F. Lorencatto, Dr R. Hunter, Dr K. Foster and Dr M. Tahir.

Funding disclosures: the POPPY-Sleep sub-study (which these analyses used sleep data collected from) was funded by National Heart Lung and Blood Institute (R01 HL131049). The parent POPPY Study waves 1 to 3 was funded by investigator-initiated grants from BMS, Gilead Sciences, Janssen, Merck and ViiV Healthcare (EudraCT Number: 2012- 003581-40; Sponsor Protocol Number: CRO1992). The POPPY study waves 4 and 5 is funded by investigator-initiated grants from Gilead Sciences, ViiV Healthcare and Merck Sharp & Dohme (UK) Limited (Sponsor protocol number: 19SM5112). The study is also supported by the National Institute for Health Research (NIHR) Biomedical Research Centre based at Imperial College Healthcare NHS Trust and Imperial College London and by an NIHR Senior Investigator Award to Professor C.A. Sabin. This material is also the result of work supported with resources and the use of facilities at the Minneapolis Veterans Affairs Medical Center, Minneapolis, USA. L.S. was funded through the National Institute for Health and Care Research Health Protection Research Unit (NIHR HPRU) in Blood Borne and Sexually Transmitted Infections at University College London in partnership with the UK Health Security Agency (HPRU Grant no: NIHR200911). The views expressed are those of the author(s) and not necessarily those of the NHS, the NIHR, the Department of Health or the funders.

### Conflicts of interest

C.S. has received funding from Gilead Sciences, ViiV Healthcare, MSD and Janssen-Cilag for membership of Advisory Boards and for preparation of educational materials. K.M.K. has received consultancy fees from Allergan/AbbVie and Data and Safety Monitoring Board activity fees from Nuvaira and Organicell, outside the work presented here. A.W. has been an investigator on studies sponsored by, received research grants from and received speaker fees or honoraria from ViiV Healthcare, Janssen, Gilead Sciences and MSD. F.A.P. reports grants and personal fees from Gilead Sciences, ViiV Healthcare and MS; all outside of the work reported here. P.W.G.M. has received honoraria and/or travel grants from Gilead Sciences, MSD, Bristol-Myers Squibb, and ViiV Healthcare, and has been awarded grants by Science Foundation Ireland, outside the submitted work. J.A. reports personal fees from Gilead Sciences and ViiV; all outside of the work reported here. M.B. has acted as a speaker or adviser to, has been an investigator for, or has received grants to her institution from Gilead, ViiV, Janssen, B.M.S., Teva, Cipla, Mylan, and MSD; all outside the work presented here. J.V. reports travel, research grants, and personal fees from Merck, Janssen Cilag, Piramal Imaging, ViiV Healthcare, and Gilead; all outside the work presented here. The funders were not involved in the study design, collection, analysis, interpretation of data, the writing of this article or the decision to submit it for publication. All authors declare no other competing interests.

## Supplementary Material

Supplemental Digital Content
