# Supplementary material for: Associations between multimorbidity burden and objective and patient-reported sleep outcomes among people with HIV
Source: AIDS. 2024 Nov 28;39(4):424–33. doi: 10.1097/QAD.0000000000004073 (PMC11872264; doi:10.1097/QAD.0000000000004073)
Supplement: Supplemental Digital Content [file aids-39-424-s001.docx]

**Associations between multimorbidity burden and objective and patient-reported sleep outcomes among people living with HIV**

**SUPPLEMENTARY MATERIAL**

**Supplementary Table 1.** List of 70 comorbidities (from 19 organ system/pathogenic groups) considered in the principal component analysis (PCA), with a prevalence >1.5% in all POPPY participants with HIV (n=1,073) at baseline

| **Organ system** | **Comorbidities** | **N (%)** |
| --- | --- | --- |
| AIDS events | AIDS-related cancers | 19 (1.8) |
|  | CMV | 28 (2.6) |
|  | Kaposi's sarcoma | 70 (6.5) |
|  | Other AIDS events | 113 (10.5) |
|  | PCP | 96 (8.9) |
|  | Tuberculosis | 78 (7.3) |
| Haematological | Anaemia | 25 (2.3) |
|  | DVT | 22 (2.1) |
| Respiratory | Asthma/bronchitis/COPD | 263 (24.5) |
|  | Hayfever | 85 (7.9) |
|  | Chest infections | 115 (10.7) |
|  | Pneumonia | 53 (4.9) |
| Cancer | Haematological cancer | 13 (1.2) |
|  | Skin cancer | 45 (4.2) |
|  | Solid organ cancer | 55 (5.1) |
| Cardiovascular | Arrhythmia | 38 (3.5) |
|  | CABG | 24 (2.2) |
|  | Dyslipidemia | 304 (28.3) |
|  | Heart failure | 23 (2.1) |
|  | Hypertension | 245 (22.8) |
|  | IHD | 46 (4.3) |
|  | Myocardial infarction | 37 (3.4) |
|  | Peripheral vascular disease | 20 (1.9) |
|  | CVA/TIA | 37 (3.4) |
| Mental health | Anxiety/Panic attacks | 96 (8.9) |
|  | Clinical depression | 346 (32.2) |
|  | Depressive symptoms | 84 (7.8) |
|  | Psychosis | 18 (1.7) |
|  | Sleeping problems | 76 (7.1) |
| Ear problems | Ear dysfunction | 61 (5.7) |
| Endocrine | Type II diabetes | 56 (5.2) |
|  | Erectile dysfunction | 78 (7.3) |
|  | Hypogonadism | 42 (3.9) |
|  | Lipodystrophy | 27 (2.5) |
|  | Hypothyroidism | 26 (2.4) |
| Eye problems | Eye problems | 88 (8.2) |
| Gastrointestinal | Persistent bowel disorders | 233 (21.7) |
|  | Hepatitis A | 45 (4.2) |
|  | Hepatitis B | 199 (18.5) |
|  | Hepatitis C | 101 (9.4) |
|  | Hernia | 42 (3.9) |
|  | Liver problems | 76 (7.1) |
|  | GORD | 82 (7.6) |
| Genitourinary | Urinary incontinence | 43 (4) |
|  | Kidney stones | 31 (2.9) |
|  | Prostate dysfunction | 37 (3.4) |
|  | Renal problems | 54 (5) |
|  | UTI | 34 (3.2) |
| Infections | Fungal infections | 52 (4.8) |
|  | HSV/VZV | 90 (8.4) |
| Joint and bone | Aches and pains | 131 (12.2) |
|  | Joint inflammation/Arthritis | 192 (17.9) |
|  | Joint replacement | 25 (2.3) |
|  | Osteopenia/osteoporosis | 85 (7.9) |
| Neurological | Encephalitis | 17 (1.6) |
|  | Epilepsy | 43 (4) |
|  | Loss consciousness | 31 (2.9) |
|  | Migraine/headaches | 52 (4.8) |
|  | Peripheral neuropathy | 238 (22.2) |
|  | Dizziness/vertigo | 116 (10.8) |
| Skin | Eczema | 115 (10.7) |
|  | Pruritus | 21 (2) |
|  | Psoriasis | 49 (4.6) |
| STDs | Chlamydia | 296 (27.6) |
|  | Gonorrhoea | 458 (42.7) |
|  | HPV | 105 (9.8) |
|  | HSV | 155 (14.4) |
|  | LGV | 45 (4.2) |
|  | Syphilis | 327 (30.5) |
| Vitamin deficiency | Vitamin D deficiency | 24 (2.2) |

**Supplementary Table 2.** Comparison of baseline socio-demographic, lifestyle and HIV-related characteristics between the final analytic sample (n=309) and those excluded from the overall POPPY study sample (n=764)

| **Characteristic** n (%) or median (IQR) | **Excluded sample** (n=764) | **Final sample** (n=309) |
| --- | --- | --- |
| Age (years) | 52 (46 – 59) | 53 (47 – 59) |
| Gender |  |  |
| Male | 648 (84.8) | 266 (86.1) |
| Female | 116 (15.2) | 43 (13.9) |
| Ethnicity |  |  |
| Black-African | 136 (17.8) | 35 (11.3) |
| White | 628 (82.2) | 274 (88.7) |
| Sexual orientation |  |  |
| MSM | 573 (75.0) | 243 (78.6) |
| Heterosexual | 191 (25.0) | 66 (21.4) |
| BMI (kg/m^2^) | 25.7 (23.5 – 28.3) | 24.8 (22.7 – 28.2) |
| Smoking status |  |  |
| Never | 321 (42.0) | 118 (38.2) |
| Past | 253 (33.1) | 112 (36.3) |
| Current | 190 (24.9) | 79 (25.6) |
| Alcohol use |  |  |
| Never | 74 (9.7) | 16 (5.2) |
| Past | 81 (10.6) | 44 (14.2) |
| Current | 609 (79.7) | 249 (80.6) |
| PHQ-9 score* | 4 (1 – 9) | 5 (1 – 10) |
| HIV-related factors |  |  |
| Years since HIV diagnosis | 12.9 (7.8 – 19.5) | 14.6 (8.1 – 21.4) |
| On ART | 746 (97.6) | 300 (97.1) |
| Current CD4+ T-cell count | 640 (479 – 823) | 602 (470 – 792) |
| *PHQ-9 scores are based on n=691 (excluded sample) vs 284 (final sample) | | |

**Supplementary Table 3.** Associations between multimorbidity burden z-scores and the sleep outcomes, adjusting for age, sex, race, obesity, sex between men, smoking status, alcohol use, history of injection drug use, recreational drug use, and without (*Model 1, n=309*) or with (*Model 2, n=284*) PHQ-9 scores. Odds ratio (OR) or Beta estimates (β), with 95% confidence intervals (CIs) are presented.

| **Follow-up**  **Burden z-scores** | **Sleep outcomes** | | | | | |
| --- | --- | --- | --- | --- | --- | --- |
|  | **Insomnia (>15 ISI score)** OR (95% confidence interval) | **Insomnia(cont.)** β (95% confidence interval) | **Sleep-Related Impairment  T-score**  β (95% confidence interval) | **Sleep Disturbance  T-score** β (95% confidence interval) | **Oxygen Desaturation Index (4% Desaturation)  (events per hour)**  β (95% confidence interval) | **Percentage of time with SpO2 below 90%**  β (95% confidence interval) |
| ***CVDs*** |  |  |  |  |  |  |
| Model 1 | 1.02 (0.99 - 1.05) P=0.19 | 1.09 (1.00 - 1.18) P=0.04 | 1.12 (1.00 - 1.25) P=0.04 | 1.05 (0.95 - 1.17) P=0.33 | 1.08 (1.00 - 1.17) P=0.05 | 1.25 (1.06 - 1.47) P=0.01 |
| Model 2 | 1.02 (0.98 - 1.05) P=0.38 | 1.06 (0.98 - 1.14) P=0.15 | 1.10 (0.99 - 1.22) P=0.09 | 1.01 (0.91 - 1.12) P=0.84 | 1.10 (1.01 - 1.19) P=0.03 | 1.29 (1.10 - 1.51) P<0.001 |
| ***Metabolic*** |  |  |  |  |  |  |
| Model 1 | 1.00 (0.97 - 1.03) P=0.94 | 1.07 (0.99 - 1.15) P=0.10 | 1.16 (1.05 - 1.29) P<0.001 | 1.13 (1.02 - 1.24) P=0.02 | 1.12 (1.04 - 1.20) P<0.001 | 1.24 (1.06 - 1.44) P=0.01 |
| Model 2 | 0.98 (0.95 - 1.01) P=0.23 | 1.00 (0.93 - 1.07) P=0.99 | 1.05 (0.95 - 1.16) P=0.31 | 1.05 (0.95 - 1.15) P=0.33 | 1.10 (1.02 - 1.19) P=0.02 | 1.18 (1.02 - 1.36) P=0.03 |
| ***Mental/Joint*** |  |  |  |  |  |  |
| Model 1 | 1.06 (1.03 - 1.09) P<0.001 | 1.20 (1.12 - 1.28) P<0.001 | 1.34 (1.22 - 1.48) P<0.001 | 1.27 (1.16 - 1.39) P<0.001 | 1.01 (0.94 - 1.08) P=0.84 | 1.09 (0.94 - 1.26) P=0.25 |
| Model 2 | 1.02 (0.99 - 1.06) P=0.14 | 1.05 (0.98 - 1.13) P=0.16 | 1.13 (1.03 - 1.25) P=0.01 | 1.10 (1.00 - 1.21) P=0.04 | 1.00 (0.93 - 1.08) P=0.97 | 1.01 (0.87 - 1.17) P=0.92 |
| ***Neurological*** |  |  |  |  |  |  |
| Model 1 | 1.01 (0.99 - 1.04) P=0.28 | 1.07 (1.00 - 1.15) P=0.05 | 1.20 (1.09 - 1.32) P<0.001 | 1.09 (1.00 - 1.19) P=0.05 | 1.02 (0.96 - 1.09) P=0.51 | 0.89 (0.77 - 1.02) P=0.10 |
| Model 2 | 0.99 (0.96 - 1.02) P=0.50 | 1.01 (0.95 - 1.08) P=0.75 | 1.11 (1.02 - 1.21) P=0.02 | 1.02 (0.93 - 1.11) P=0.70 | 1.00 (0.93 - 1.07) P=0.95 | 0.88 (0.77 - 1.01) P=0.06 |
| ***Cancer/Other*** |  |  |  |  |  |  |
| Model 1 | 0.97 (0.94 - 1.00) P=0.06 | 0.96 (0.89 - 1.03) P=0.25 | 0.98 (0.88 - 1.09) P=0.70 | 0.98 (0.89 - 1.08) P=0.64 | 1.00 (0.93 - 1.08) P=0.97 | 1.15 (0.98 - 1.34) P=0.08 |
| Model 2 | 0.93 (0.89 - 0.97) P<0.001 | 0.92 (0.86 - 0.99) P=0.02 | 0.92 (0.83 - 1.01) P=0.07 | 0.94 (0.86 - 1.03) P=0.20 | 1.00 (0.93 - 1.08) P=0.93 | 1.06 (0.91 - 1.23) P=0.44 |

**Supplementary Table 4.** Univariable and multivariable models showing the associations between changes in multimorbidity burden z-scores and the sleep outcomes. The multivariate model adjusted for age, sex, race, obesity, sex between men, smoking status, alcohol use, history of injection drug use and recreational drug use. Odds ratio (OR) or Beta estimates (β), with 95% confidence intervals (CIs) are presented.

| **Change in burden z-scores** | **Sleep outcomes** | | | | | |
| --- | --- | --- | --- | --- | --- | --- |
|  | **Insomnia (>15 ISI score)** OR (95% confidence interval) | **Insomnia (cont.)** β (95% confidence interval) | **Sleep-Related Impairment  T-score**  β (95% confidence interval) | **Sleep Disturbance  T-score** β (95% confidence interval) | **Oxygen Desaturation Index (4% Desaturation)  (events per hour)**  β (95% confidence interval) | **Percentage of time with SpO2 below 90%**  β (95% confidence interval) |
| ***CVDs*** |  |  |  |  |  |  |
| Unadjusted | 1.01 (0.95 - 1.08) P=0.74 | 1.08 (0.90 - 1.28) P=0.41 | 1.25 (0.98 - 1.60) P=0.07 | 1.14 (0.91 - 1.44) P=0.25 | 0.99 (0.83 - 1.19) P=0.94 | 1.44 (0.99 - 2.10) P=0.06 |
| Adjusted | 1.01 (0.94 - 1.08) P=0.79 | 1.06 (0.89 - 1.27) P=0.51 | 1.24 (0.97 - 1.59) P=0.09 | 1.13 (0.90 - 1.43) P=0.30 | 0.95 (0.80 - 1.13) P=0.56 | 0.93 (0.66 - 1.32) P=0.68 |
| ***Metabolic*** |  |  |  |  |  |  |
| Unadjusted | 0.99 (0.90 - 1.08) P=0.75 | 0.91 (0.73 - 1.14) P=0.43 | 1.03 (0.75 - 1.42) P=0.84 | 0.90 (0.66 - 1.21) P=0.47 | 1.01 (0.80 - 1.27) P=0.92 | 1.42 (0.87 - 2.31) P=0.16 |
| Adjusted | 1.01 (0.92 - 1.11) P=0.86 | 0.94 (0.75 - 1.19) P=0.63 | 1.11 (0.80 - 1.54) P=0.52 | 0.92 (0.68 - 1.26) P=0.62 | 0.91 (0.73 - 1.14) P=0.42 | 1.09 (0.70 - 1.71) P=0.70 |
| ***Mental/Joint*** |  |  |  |  |  |  |
| Unadjusted | 1.09 (1.03 – 1.17) P=0.01 | 1.38 (1.14 - 1.67) P<0.001 | 1.87 (1.43 - 2.43) P<0.001 | 1.53 (1.18 - 1.97) P<0.001 | 0.92 (0.76 - 1.12) P=0.43 | 0.95 (0.63 - 1.45) P=0.82 |
| Adjusted | 1.09 (1.02 - 1.17) P=0.01 | 1.37 (1.13 - 1.67) P<0.001 | 1.78 (1.35 - 2.33) P<0.001 | 1.48 (1.14 - 1.93) P<0.001 | 0.96 (0.79 - 1.17) P=0.67 | 1.02 (0.68 - 1.53) P=0.92 |
| ***Neurological*** |  |  |  |  |  |  |
| Unadjusted | 1.00 (0.95 – 1.05) P=0.91 | 0.98 (0.85 - 1.12) P=0.77 | 1.10 (0.91 - 1.33) P=0.34 | 0.92 (0.77 - 1.11) P=0.39 | 1.10 (0.95 - 1.26) P=0.20 | 0.92 (0.68 - 1.23) P=0.57 |
| Adjusted | 0.99 (0.94 - 1.05) P=0.83 | 0.99 (0.87 - 1.14) P=0.94 | 1.12 (0.92 - 1.36) P=0.25 | 0.93 (0.77 - 1.12) P=0.43 | 1.08 (0.94 - 1.24) P=0.26 | 0.84 (0.62 - 1.12) P=0.23 |
| ***Cancer/Other*** |  |  |  |  |  |  |
| Unadjusted | 1.00 (0.95 – 1.06) P=0.89 | 0.99 (0.84 - 1.16) P=0.86 | 1.06 (0.85 - 1.32) P=0.61 | 1.09 (0.89 - 1.35) P=0.39 | 1.03 (0.88 - 1.21) P=0.73 | 1.09 (0.78 - 1.52) P=0.63 |
| Adjusted | 1.00 (0.95 - 1.07) P=0.88 | 0.97 (0.83 - 1.14) P=0.72 | 1.04 (0.83 - 1.30) P=0.73 | 1.04 (0.85 - 1.29) P=0.68 | 1.06 (0.91 - 1.24) P=0.44 | 0.98 (0.72 - 1.33) P=0.89 |

**Supplementary Table 5.** All comorbidities with a correlation >0.40 with corresponding PCs or patterns

| PC (% of variance explained) | Label | Comorbidities with correlation >0.25 (correlation with PC) |
| --- | --- | --- |
| 1 (6.5%) | *CVDs* | CABG/PCTA (0.68), Heart failure (0.62), Hypertension (0.70), IHD (0.72), Myocardial infarction (0.69), Peripheral vascular disease (0.46), Renal problems (0.40) |
| 2 (4.5%) | *STDs* | Gonorrhoea (0.78), Chlamydia (0.68), LGV (0.64), Syphilis (0.64), HSV (0.48) |
| 3 (3.8%) | *Metabolic* | Peripheral neuropathy (0.57), Type II diabetes (0.57), Hypothyroidism (0.46) Dyslipidemia (0.45), Pruritis (0.41) |
| 4 (3.1%) | *Mental/Joint* | Clinical depression (0.75), Anxiety/Panic attacks (0.50), Joint inflammation/ Arthritis (0.45), Joint replacement (0.45), Sleeping problems (0.40), Bowel disorders (0.40) |
| 5 (2.9%) | *Neurological* | Dizziness/Vertigo (0.61), Encephalitis (0.60), Loss of consciousness (0.40) |
| 6 (2.6%) | *Cancer/Other* | Haematological cancer (0.64), Hernia (0.45), Osteopenia/osteoporosis (0.44), AIDS-related cancer (0.43) |
| CVDs; Cardiovascular diseases, CABG/PCTA; coronary-artery bypass grafting/percutaneous transluminal coronary angioplasty, IHD; Ischemic heart disease, STDs; Sexually transmitted diseases, LGV; Lymphogranuloma venereum, HSV; Herpes simplex virus | | |

**Supplementary Table 6.** Univariable and multivariable models showing the associations of morbidity burden z-scores, based on a PCA threshold >0.40, with sleep outcomes using logistic/linear regression models; reported estimates show the estimated change in the odds ratio (insomnia, ISI>15) and score (ISI, PROMIS-SRI, PROMIS-SD, ODI and percentage of time with Spo2 below 90%) associated with a 0.1 standard deviation increase in each burden z-score

| **Morbidity burden z-scores** | **Sleep outcomes** | | | | | |
| --- | --- | --- | --- | --- | --- | --- |
|  | **Insomnia (>15 ISI score)** OR (95% confidence interval) | **Insomnia (cont.)** β (95% confidence interval) | **Sleep-Related Impairment  T-score**  β (95% confidence interval) | **Sleep Disturbance  T-score** β (95% confidence interval) | **Oxygen Desaturation Index (4% Desaturation)  (events per hour)**  β (95% confidence interval) | **Percentage of time with SpO2 below 90%**  β (95% confidence interval) |
| ***CVDs*** |  |  |  |  |  |  |
| Unadjusted | 1.01 (0.98 - 1.04) p=0.39 | 1.03 (0.96 - 1.11) p=0.40 | 1.05 (0.94 - 1.16) p=0.41 | 1.00 (0.90 - 1.10) p=0.95 | 1.11 (1.03 - 1.20) p=0.01 | 1.28 (1.09 - 1.50) p<0.001 |
| Adjusted | 1.01 (0.98 - 1.04) p=0.44 | 1.06 (0.97 - 1.15) p=0.18 | 1.08 (0.96 - 1.21) p=0.19 | 1.04 (0.93 - 1.16) p=0.46 | 1.04 (0.96 - 1.12) p=0.39 | 1.12 (0.95 - 1.32) p=0.16 |
| ***Metabolic*** |  |  |  |  |  |  |
| Unadjusted | 1.01 (0.98, 1.03) p=0.65 | 1.07 (1.00 - 1.15) p=0.05 | 1.10 (1.00 - 1.22) p=0.05 | 1.14 (1.04 - 1.25) p=0.01 | 1.00 (0.93 - 1.07) p=0.94 | 1.27 (1.10 - 1.48) p<0.001 |
| Adjusted | 1.01 (0.98 - 1.04) p=0.62 | 1.08 (1.00 - 1.16) p=0.04 | 1.12 (1.01 - 1.24) p=0.03 | 1.13 (1.03 - 1.25) p=0.01 | 0.99 (0.92 - 1.06) p=0.69 | 1.11 (0.96 - 1.29) p=0.15 |
| ***Mental/Joint*** |  |  |  |  |  |  |
| Unadjusted | 1.02 (0.99 - 1.04) p=0.26 | 1.04 (0.97 - 1.11) p= 0.30 | 1.14 (1.03 - 1.26) p=0.01 | 1.06 (0.96 - 1.17) p=0.23 | 1.09 (1.02 - 1.17) p=0.02 | 1.19 (1.02 - 1.38) p=0.03 |
| Adjusted | 1.02 (0.99 - 1.05) p=0.21 | 1.04 (0.96 - 1.12) p=0.35 | 1.13 (1.02 - 1.25) p=0.02 | 1.04 (0.94 - 1.15) p=0.42 | 1.08 (1.00 - 1.16) p=0.04 | 1.05 (0.91 - 1.22) p=0.50 |
| ***Neurological*** |  |  |  |  |  |  |
| Unadjusted | 1.01 (0.98 - 1.04) p=0.58 | 1.05 (0.97 - 1.13) p= 0.23 | 1.16 (1.05 - 1.29) p<0.001 | 1.14 (1.03 - 1.25) p=0.01 | 0.96 (0.89 - 1.04) p=0.31 | 1.02 (0.87 - 1.20) p=0.77 |
| Adjusted | 1.01 (0.98 - 1.04) p=0.47 | 1.05 (0.97 - 1.13) p=0.26 | 1.15 (1.03 - 1.28) p=0.01 | 1.12 (1.01 - 1.24) p=0.03 | 0.96 (0.89 - 1.04) p=0.30 | 1.06 (0.91 - 1.24) p=0.45 |
| ***Cancer/Other*** |  |  |  |  |  |  |
| Unadjusted | 0.99 (0.96 - 1.02) p=0.49 | 1.03 (0.96 - 1.11) p=0.40 | 1.12 (1.01 - 1.24) p=0.03 | 1.08 (0.98 - 1.19) p=0.13 | 1.02 (0.94 - 1.09) p=0.66 | 1.07 (0.91 - 1.25) p=0.41 |
| Adjusted | 0.99 (0.96 - 1.02) p=0.45 | 1.03 (0.95 - 1.11) p=0.53 | 1.12 (1.01 - 1.25) p=0.03 | 1.07 (0.97 - 1.19) p=0.18 | 1.00 (0.92 - 1.07) p=0.90 | 1.02 (0.87 - 1.18) p=0.84 |
